# Antioxidant-enriched autologous biogel promoted diabetic wound healing by remodeling inherent posttraumatic inflammatory patterning and restoring compromised microenvironment homeostasis

**DOI:** 10.1093/rb/rbac023

**Published:** 2022-04-29

**Authors:** Yixi Yang, Le Wang, Yonglin Zhou, Yijun He, Shaozhang Lin, Yuwei Zeng, Yunhe Zhou, Wei Li, Zaopeng He, Qi Zhao, Lihao Chen, Zijie Li, Wenhao Wang, Zhi-Yong Zhang

**Affiliations:** 1 Translational Research Centre of Regenerative Medicine and 3D Printing of Guangzhou Medical University, Guangdong Province Engineering Research Center for Biomedical Engineering, State Key Laboratory of Respiratory Disease, The Third Affiliated Hospital of Guangzhou Medical University, Guangzhou 510150, P. R. China; 2 Department of Orthopaedic Surgery, The Third Affiliated Hospital of Guangzhou Medical University, Guangzhou 510150, P. R. China; 3 Medical Technology and Related Equipment Laboratory, The Third Affiliated Hospital of Guangzhou Medical University, Guangzhou 510150, P. R. China; 4 Hand and Foot Surgery & Plastic Surgery, Affiliated Shunde Hospital of Guangzhou Medical University, Shunde District, Foshan, P. R. China

**Keywords:** autologous regeneration factors, wound healing, homeostasis, tissue microenvironment, inflammation patterning

## Abstract

Successful wound healing depends on the reconstruction of proper tissue homeostasis, particularly in the posttraumatic inflammatory tissue microenvironment. Diabetes jeopardizes tissues’ immune homeostasis in cutaneous wounds, causing persistent chronic inflammation and cytokine dysfunction. Previously, we developed an autologous regeneration factor (ARF) technology to extract the cytokine composite from autologous tissue to restore immune homeostasis and promote wound healing. However, treatment efficacy was significantly compromised in diabetic conditions. Therefore, we proposed that a combination of melatonin and ARF, which is beneficial for proper immune homeostasis reconstruction, could be an effective treatment for diabetic wounds. Our research showed that the utilization of melatonin-mediated ARF biogel (AM gel) promoted diabetic wound regeneration at a more rapid healing rate. RNA-Seq analysis showed that AM gel treatment could restore more favorable immune tissue homeostasis with unique inflammatory patterning as a result of the diminished intensity of acute and chronic inflammation. Currently, AM gel could be a novel and promising therapeutic strategy for diabetic wounds in clinical practice through favorable immune homeostatic reconstructions in the tissue microenvironment and proper posttraumatic inflammation patterning.

## Graphical Abstract

**Figure:**
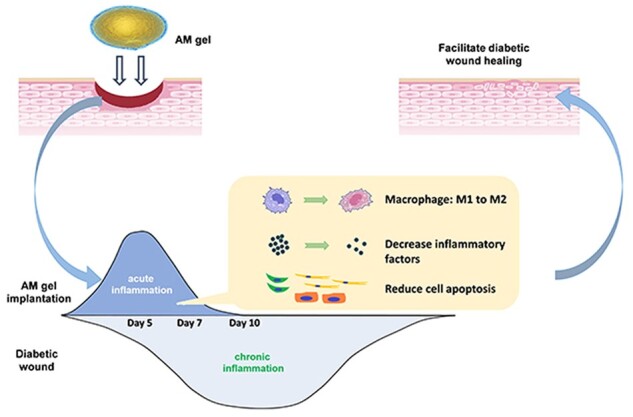

## Introduction

Diabetes mellitus is a group of metabolic diseases characterized by hyperglycemia resulting from insulin dysfunction [1], in which tissue regeneration is suppressed [2]. Patients with diabetes, particularly the elderly, suffer from a series of complications that lead to tissue damage [3]. Delayed wound healing remains one of the most severe diabetic complications in elderly patients, leading to delayed daily activity and health span, or even amputation [4]. The treatment of delayed diabetic wound healing is a major challenge in clinical management, particularly with the increasing diabetic patient population in an aging society. Despite the emergence of basic research, existing clinical interventions are limited and generally ineffective.

Wound healing requires the appropriate integration of complex biological and molecular processes, such as cell function and proliferation [5]; however, the posttraumatic inflammatory patterning observed in diabetic wounds caused by advanced glycation end products is prolonged and persists [6]. Compromised immune tissue homeostasis, which is characterized by chronic and prolonged activation of posttraumatic inflammation, is a hallmark of non-healing diabetic wounds [7]. Therefore, several studies have considered anti-inflammatory approaches to be an appropriate treatment for diabetic refractory wounds. In a recent study, researchers optimized the inflammatory microenvironment using interleukin-4 (IL-4) as an anti-inflammatory factor and confirmed its potential in regulating inflammation [8]. The cytokine mixture comprising both interleukin-10 (IL-10) and prostaglandin-E2 was designed to reduce inflammatory reactions by inducing macrophages into the M2 prohealing phenotype [9]. Nevertheless, supplementary exogenous cytokines are limited for use in clinical practice due to their high cost and heterogeneous origin.

An inherent cytokine composite is crucial for homeostatic and cytokine-mediated inflammation patterning, which plays a pivotal role in restoring homeostasis [10] and tissue microenvironment homeostasis affects the ability of traumatic tissue regeneration. Homeostatic imbalance causes abnormalities in various cytokines, eventually leading to cell apoptosis and persistent dysfunctional and pathological states [11]. In our previous research, a complete process technology, which was developed based on the extraction of inherent cytokine composites derived from platelets [12], adipose tissue [13] or other autologous tissue, restored homeostasis by modulating inflammatory patterning. The therapeutic effects of autologous regeneration factors (ARFs) have been attributed to the appropriate cytokine composition and concentration, leading to inflammatory regulation, homeostatic balance restoration and, eventually, tissue regeneration. ARF is more convenient and safe to use due to its autologous source, one preparation-repeated use and multiple dosage forms that can be designed for clinical application. However, during the treatment of diabetic wounds, we found that ARF therapy had limited efficacy.

The process of diabetic repair is impeded by the compromised microenvironment caused by hyperglycemia [14]. The excessive accumulation of reactive oxygen species (ROS) in diabetic wounds results in the destruction of microenvironmental homeostasis and hinders wound tissue regeneration [15]. Such immunomodulation and ROS scavenging can be accomplished by releasing anti-inflammatory agents [16]. Moreover, the extracted compromising cytokine in the diabetic microenvironment also affects the quality of treatment for ARF. In previous studies, melatonin has been considered an antioxidant that promotes diabetic wound healing via immune regulation [17]. Melatonin is of particular concern because some experiments have indicated that melatonin suppresses the prolonged inflammatory phase through ROS scavenging and anti-inflammatory effects [18], which is usually used in clinical treatment as a preventive therapy for certain diseases [19]. Melatonin is universally important for the human body [20] as it exhibits protective effects in both physiological and pathological states [21]. Recent research has demonstrated that melatonin can promote epithelial cell proliferation [22] and facilitate wound healing [23]. Dynamic changes in the phenotype of macrophages lead to differences in wound healing [24] and certain studies have suggested that melatonin induces M1–M2 phenotype transformation in macrophages [18].

The immune tissue homeostasis is compromised in diabetic cutaneous wounds, which are characterized by persistent chronic inflammation and present an inherent pattern of posttraumatic inflammation. Limited targeted research is available on the continuous variation of inflammation, namely inflammation patterning after trauma in diabetes. The remodeling of this inherent patterning may reveal the need for a novel therapeutic method for accelerating tissue repair. Considering the key functions of melatonin and ARF, we hypothesized that the application of melatonin-enriched ARF biogel (AM gel) treatment may promote diabetic wound healing by remodeling the inherent posttraumatic inflammation patterning and restoring immune homeostasis in the tissue microenvironment. In the present study, we excised the skin of the dorsal trunk of rats with Type I diabetes mellitus to establish a delayed healing wound model from persistent inflammation and oxidative stress [25]. After treatment, the results proved that accurate and proactive immunomodulation via AM gel introduces a semblance of acute inflammatory response to initiate diabetic wound healing. Subsequently, excessive inflammatory expression diminishes to restore cell homeostasis and accelerate healing. More importantly, platelet concentrates are routinely used in clinical practice [26], while the anti-inflammatory, antioxidant and mitochondrial protective effects of melatonin have also been documented in multiple clinical trials [27–29]. This novel therapeutic strategy demonstrates a promising and feasible intervention for clinical applications.

## Materials and methods

### Materials

Melatonin, fetal bovine serum (FBS) and trypsin-EDTA were purchased from Sigma-Aldrich (St. Louis, MO, USA). Dulbecco’s modified Eagle’s medium (DMEM) and penicillin–streptomycin were purchased from Gibco Life Technologies (Grand Island, NY, USA). The antibodies of advanced glycosylation end products (AGEs) of bovine serum albumin (AGEs-BSA), primary antibodies against CD68 (ab283654), CD86 (ab239075), nitric oxide synthase 2 (iNOS2, ab283655) and ARG (arginase Type II, ab264066) were purchased from Abcam (Cambridge, UK). PE anti-mouse CD86 (159204) and FITC anti-mouse CD206 (141703) were purchased from BioLegend (San Diego, CA, USA), and alpha-smooth muscle actin (α-SMA) (BM0002) was purchased from Boster Biological Technology (Wuhan, China). Masson’s Staining Kit (G1340) was purchased from Solarbio (Beijing, China).

### Autologous regeneration factors

ARFs were isolated from 36 ml of human peripheral blood collected from healthy volunteers. All blood samples were collected in blood collection tubes (9 ml) containing 3.2% sodium citrate anticoagulant, and were further vigorously mixed by rotating 180° upside-down eight times. ARFs were prepared via two-step centrifugation (400 g, 10 min): (i) The bottom layer of red blood cells was removed after the first centrifugation. (ii) After the second centrifugation, the upper pure plasma was removed to another vial for collection, and the residual compounds (platelet-concentrated plasma) were mixed and transferred to a vial containing thrombin–CaCl_2_ solution (thrombin and calcium chloride, 5 ml, 5% calcium chloride solution to 2000 U thrombin) at 37°C for 1 h to release the ARF [30], the ratio of thrombin–CaCl_2_ solution and platelet-concentrated plasma were 1:10 (v/v). The released ARF and the collected plasma were then pre-frozen at −80°C for 24 h before being freeze-dried for 48 h using a lyophilizer. After lyophilizing, the ARF was stored at −20°C [31].

### Treatment of animals

#### Preparation of rat ARF and AM gel

Male rats (aged 7 weeks old) were purchased from Guangdong Medical Laboratory Animal Center, China. Rat ARF was manufactured from 9 ml rat arterial blood extracted from the aorta ventralis of Sprague Dawley (SD) rats. Some of the SD rats were anesthetized with 2% sodium pentobarbital (40 mg/kg), underwent abdominal aortic puncture and blood samples were collected in tubes (9 ml) containing 3.2% sodium citrate anticoagulant. The rat ARF was created via a two-step centrifugation process and activated by mixing thrombin–CaCl_2_ with ARF, which is similar to the preparation of human ARF. Rat ARF and rat plasma were pre-frozen at −80°C for 24 h before being freeze-dried for 48 h using a lyophilizer. After lyophilizing, the ARF was stored at −20°C before use. After dissolving the lyophilized ARF and plasma powder in 20 μM melatonin containing phosphate buffer saline (PBS), respectively, the total dosage of PBS (the volume of ARF plus plasma before freeze-drying) was consistent with that before freeze-drying. Their compounds formed hydrogels (AM gel) during application, which were mediated by the interaction between the thrombin in the ARF powder and the fibrinogen in the ungelated plasma powder, and the concentration of melatonin in the AM gel was 20 μM (4.65 μg/ml). All animal experiments were approved by the Animal Ethics Committee of the Guangdong Medical Laboratory Animal Center.

#### Properties of AM gel

Rheological test: AM gels were prepared as disks (25 mm diameter and 0.2 mm thickness) with a special mold. An Anton Paar MCR-301 rheometer equipped with a 25 mm parallel plate was used for the rheological test. A frequency sweep between 0.1 and 25 rad/s at 20°C was performed. Three independent samples were measured, and the data were reported as mean ± standard deviations.

Compression test: AM gel was blotted dry and placed on a cylinder plate of a DAM Q800 mechanical tester after gelation. The sample was compressed at a constant speed of 0.5 N/min and load cell of 18 N at room temperature. The compressive modulus was calculated as the slope corresponding to 10–20% strain in the linear region of the stress–strain curve.

Swelling ratio test: AM gels were placed in PBS at 37°C for 24 h, taken from DPBS, dried and weighed (W_S_). Dry weights (W_D_) were determined after freeze-drying. Three independent samples were measured, and the swelling ratio (SR) was calculated according to the Equation: SR *=* (W_S_–W_D_)/W_D_ × 100%.

Degradation ratio test: AM gels were incubated in 2 ml Eppendorf tubes with 1 ml collagenase solution (PBS containing 2U/ml of collagenase Type II) at 37°C continuously for 7 days, during which the collagenase solution was refreshed every day. Gels were washed with PBS three times after removing the collagenase solution at predetermined time points (Days 3, 6, 9). Samples were freeze-dried and weighed before calculation. Three independent samples were measured, and the percentage of degradation (D%) was calculated according to Equation: D% *=* (W_0_ – W_t_)/W_0_ × 100%, W_0_ represents the initial dry weight of gels (Day 0), and W_t_ represents the dry weight of gels at each time point.

Melatonin release test: 1 ml of AM gels (melatonin concentration: 20 μM, 4.65 μg/ml) were placed in 2 ml of PBS at 37°C at different time points after gelification (6 h to 7 days). PBS was collected for analysis at each time point, and was replaced with a new 2 ml of PBS. Three independent samples were measured using enzyme-linked immunosorbent assays to quantify melatonin. The collected PBS were diluted, and the absorption was detected at 450–560 nm.

#### Streptozotocin-induced diabetes

All rats were intraperitoneally injected once with streptozotocin (STZ; 60 mg/kg, Sigma) in pH 4.5 citrate buffer. Diabetes was considered if the glucose level consistently exceeded 14 mmol/L in heparinized tail vein blood after 1 week, and these hyperglycemic rats were used in the wound healing model assay 1 week after STZ treatment.

#### *In vivo* wound healing model

**Figure 1.**
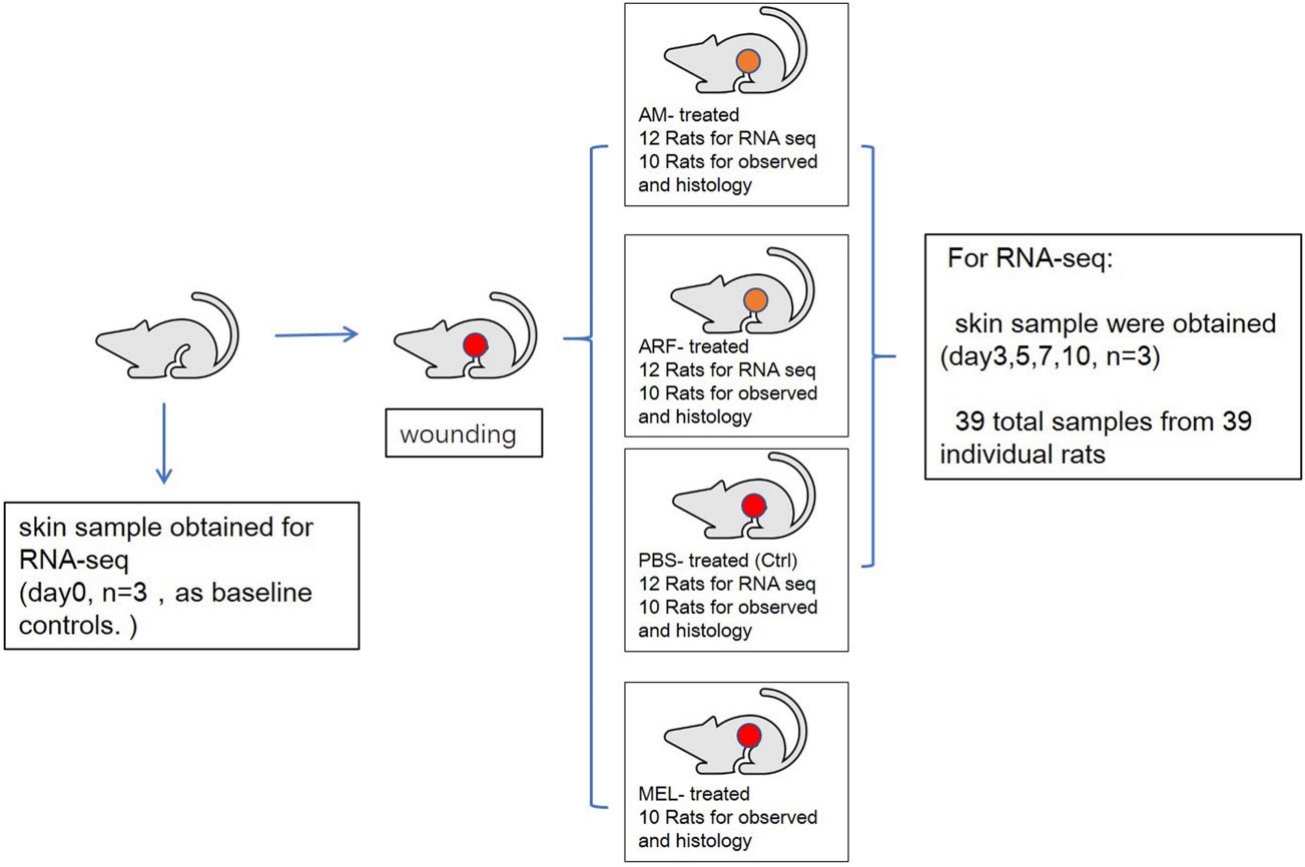
Schematic flow chart of experiment *in vivo*

Diabetic rats were randomly divided into four groups: control (*n* = 10), ARF (*n* = 10), melatonin (*n* = 10) and AM gel (ARF + melatonin) (*n* = 10) groups (Fig. 1). All rats were anesthetized with 2% sodium pentobarbital (40 mg/kg), and the dorsal area was shaved and cleaned with povidone-iodine solution and alcohol swabs. Two full-thickness dermal circular wounds of 8 mm were created on each side of the dorsal trunk with a punch using surgical scissors. Thereafter, we fixed thick silicone (0.5 mm), doughnut-shaped splints (external diameter 20 mm, internal diameter 10 mm) on each wound with 3–0 prolene sutures. In each group, the wound surfaces were covered by different types of gel with a volume of 200 μL. As for the melatonin group, 1 mM melatonin/dimethylsulfoxide (DMSO) solution was diluted to 20 μM with rat plasma, followed by gelation with thrombin–CaCl_2_. In the ARF group, the gel was prepared by mixing lyophilized ARF powder with rat plasma. Similarly, in the AM group, the gel was prepared by mixing 20 μM of melatonin containing ARF with resolubilized rat plasma to create the AM gel. The control group, covered with 200 μL of PBS on the wound, served as the control. After covering, we pasted 3M Tegaderm transparent films on the doughnut-shaped splints to confine the gels. After the different treatments, as mentioned above, all wounds were analyzed by Image J software at each time points (Days 0, 7, 14, 21), and the percentage of wound closure was quantified by comparing the pixel area of the wound with the pixel area of the image captured from the original wound at Day 0. Body weight and blood glucose concentrations were monitored after wounding over time (Supplementary Fig. S1).

#### Histological analysis

Skin tissues were collected at each time point (7, 14, 21 days) and fixed with 4% paraformaldehyde 48 h before embedding in paraffin. Paraffin sections (5 μm) of samples were stained with hematoxylin and eosin (H & E), and collagen was stained with Masson’s trichrome staining. Images were captured under an optical microscope.

#### Immunohistochemical staining

Skin tissues were obtained (as before) on Day 7 for immunofluorescence staining, and paraffin sections were deparaffinized and heated for antigen recovery using citrate buffer (pH 6.0). After washing three times with PBS, the samples were blocked with 5% BSA for 30 min and incubated with primary antibody at 4°C overnight. After further washing three times with PBS, the sections were incubated with the corresponding secondary antibodies for 50 min in the dark. Fluorescent images were captured under an inverted fluorescence microscope. Under 400 × magnification, 6–8 fields were randomly selected and the positive proportions in each field were quantitatively evaluated using ImageJ software.

#### Dihydroethidium staining

Skin tissues were obtained as aforementioned on Day 7 for dihydroethidium (DHE) staining, and were frozen by liquid nitrogen. Frozen sections were incubated in the dark with DHE, which was diluted with PBS (20 μM) at 37°C for 30 min. Fluorescent images were captured under an inverted fluorescence microscope. Under 200 × magnification, three fields were randomly selected and the DHE fluorescence intensity were quantified by ImageJ software.

#### RNA-Seq and analysis

For RNA-Seq, three samples of diabetic skin with a diameter of 1 cm were excised on Day 0 as baseline controls. A circle of 1 cm diameter around the healing wound was excised from all experimental groups for wound sampling. At different time points (Days 3, 5, 7, 10), wound samples (*n* = 3) were harvested from the AM, ARF and control groups, respectively (Fig. 1). Total RNA from each sample was isolated using TRIzol Reagent (Thermo Fisher Scientific), then quantitatively determined using a NanoDrop and sequenced by an Agilent 2100 Bioanalyzer (Thermo Fisher Scientific, MA, USA). 300 ng of total RNA per group at each time point (100 ng from each rat) was used for library preparation and sequencing on a BGISEQ–500 (BGI–Shenzhen, China). The RNA-Seq data were compared using bioinformatics tools, and the threshold was a fold change > 2 (upregulation or downregulation).

Bioinformatics analysis: The sequencing data of RNA-Seq was filtered with SOAPnuke (v 1.5.2) by removing sequencing adapter, low-quality reads and unknown bases. The clean reads were mapped to the reference genome of rats with HISAT2. Differential expression analysis of all rat genes was performed with the DESeq2, and differential genes (Q value ≤ 0.05) were annotated by gene ontology (http://www.geneontology.org/) enrichment analysis. The significant levels of terms and pathways were corrected by the Q value with a rigorous threshold (Q value ≤ 0.05).

#### Real-time polymerase chain reaction analysis

All samples were collected, and RNA was extracted as described above. The cDNA templates were synthesized from 2 μg total RNA for each sample using reverse transcriptase, according to the manufacturer’s protocol (DBI Bioscience, Germany). The mRNA levels were quantified using the ABI PRISM 7500 Sequence Detection System (ABI, Foster City, CA, USA) according to the manufacturer’s instructions. The levels of CD68, CD86, CD3e, vascular endothelial growth factor (VEGF), platelet derived growth factor (PDGF), fibroblast growth factor (FGF), transforming growth factor-β (TGF-β), collagen I and GAPDH transcripts were examined. All data were analyzed using the 2−ΔΔCt method, and the expression fold changes were normalized to the levels of GAPDH transcripts. The experiments were performed three times independently, and the primer sequences used are listed in Supplementary Table S1.

### Culture and treatment of cells

#### Culture of RAW264.7, HUVEC, L929 and Hacat

L929 dermal fibroblasts and mouse leukemia cells of monocyte macrophages (RAW264.7) were obtained from the Chinese Academy of Sciences (Shanghai, China). Human umbilical vein endothelial cells (HUVEC) were purchased from the American Type Culture Collection (USA). Human immortalized keratinocytes (Hacat) were obtained from Zhong Qiao Xin Zhou Biotechnology Co., Ltd. (Shanghai, China). All cells were cultured in DMEM containing 10% FBS, 100 U/ml penicillin and 100 μg/ml streptomycin. The cells were cultured at 37°C in 5% CO_2_ and a humidified incubator.

#### Preparation of AGEs, ARF and melatonin-treated macrophage conditioned medium

Advanced glycation end products (AGEs) are significant factors that promote inflammation and oxidative stress in diabetes mellitus, and they have been used as an *in vitro* model of the diabetic environment in many research [32]. After a 48-h culture period, conditioned medium (CM) was acquired from supernatants of RAW264.7 (2 × 10^4^ cells/well in six-well plates) cultured in DMEM (2 ml/well in six-well plates) supplemented with:

1. AGEs 100 μg/ml (AGEs-CM)
2. AGEs 100 μg/ml and ARF 1% (A-ARF-CM)
3. AGEs 100 μg/ml and melatonin 20 μM (A-MEL-CM)
4. AGEs 100 μg/ml, ARF 1%, and melatonin 20 μM (A-AM-CM)
5. Lipopolysaccharide (LPS) 100 ng/ml (LPS-CM)
6. No supplementation (Ctrl-CM).

After collection, the conditioned media were centrifuged (for 15 min at 10 000 rpm) to remove the cellular debris and were filter-sterilized through a syringe filter (0.22 μm) before being stored at −80°C. All CM were diluted in a ratio of 1:1 (v/v) with 10% FBS DMEM as a 50% CM.

#### Treatment of HUVEC, L929 and Hacat with A-AM-CM

HUVEC, L929 and Hacat were cultured with different condition media prepared at 37°C in a 5% CO_2_ humidified atmosphere. All groups of cells were subjected to cytological analyses, as mentioned below.

#### Viability assay of HUVEC, L929 and Hacat

The viability of HUVEC, L929 and Hacat was evaluated using the cell counting kit-8 (CCK-8) assay to determine the extent of cell proliferation. HUVEC, L929 and Hacat were seeded at a density of 4000 cells/well in a 96-well plate and incubated overnight. The media were removed, and thereafter, the CM of each group was added and cultured for 24 h, with five wells per group. After treatment, 10 μL of CCK-8 solution was added away from the light. After incubation for an additional 2 h, absorption was detected at 450 nm.

#### Assay of HUVEC tube formation

Matrigel solution was placed at 50 μL/well in 96-well-plates at 37°C for 2 h for solution solidification. HUVEC were placed into the wells (2 × 10^4^ cells/well) in the media containing AGEs, ARF, melatonin or different conditioned media: AGEs-CM, A-ARF-CM, A-MEL-CM and A-AM-CM at 37°C. Tube-like formations were observed after 12 h, and four independent representative fields were photographed in each well using a microscope (× 100), and the average length of the tubes was quantitatively measured using Image J software.

#### HUVEC and L929 scratch wound assays

HUVEC or L929 were cultured for 5 × 10^5^ cells/well in 6-well plates and were incubated until they formed a confluent monolayer. An artificial incisional wound was made by gently scratching in a straight line. Next, the plates were washed with PBS, and the medium containing AGEs, ARF and melatonin was added to HUVEC, whereas AGEs-CM, A-ARF-CM, A-MEL-CM and A-AM-CM were added to L929 and cultured at 37°C. All cell groups were assessed, and cell migration rates were quantitatively evaluated using ImageJ software at established time points (6 h and 12 h).

#### Flow cytometry

HUVEC were treated with or without AGEs (100 μg/ml), ARF (1%) or melatonin (20 μM), as mentioned for the *in vitro* apoptosis assay. Approximately 1 × 10^6^ cells were analyzed by the fluorescent dye annexin V-FITC/propidium iodide (PI) to detect apoptotic cells (annexin V+/PI− and annexin V+/PI+).

RAW264.7 cells were cultured to 40% confluence in DMEM containing AGEs (100 μg/ml), and treated with or without ARF (1%) and melatonin (20 μM). Moreover, certain cells were stimulated with LPS (100 ng/ml, M1 polarized) or IL-4 (40 ng/ml, M2 polarized) for activation as the positive control. Following digestion and collection, cells were stained with 1% BSA containing the FITC-conjugated anti-mouse CD86 antibody (Biolegend, 159204, USA) or PE-conjugated anti-mouse CD206 antibody (Biolegend, 141703, USA) to confirm the polarization of macrophages, respectively. All data were analyzed using a BD Accuri C6 flow cytometer.

### Statistical analysis

Data analysis was performed with GraphPad Prism 8.0 software (GraphPad Software, San Diego, CA, USA), and a *P* values of < 0.05 was considered statistically significant. The differences between groups were analyzed using one-way ANOVA followed by Tukey’s *t* test. All analyses were performed in at least three independent experiments.

## Results

### Physical properties of AM gel

**Figure 2.**
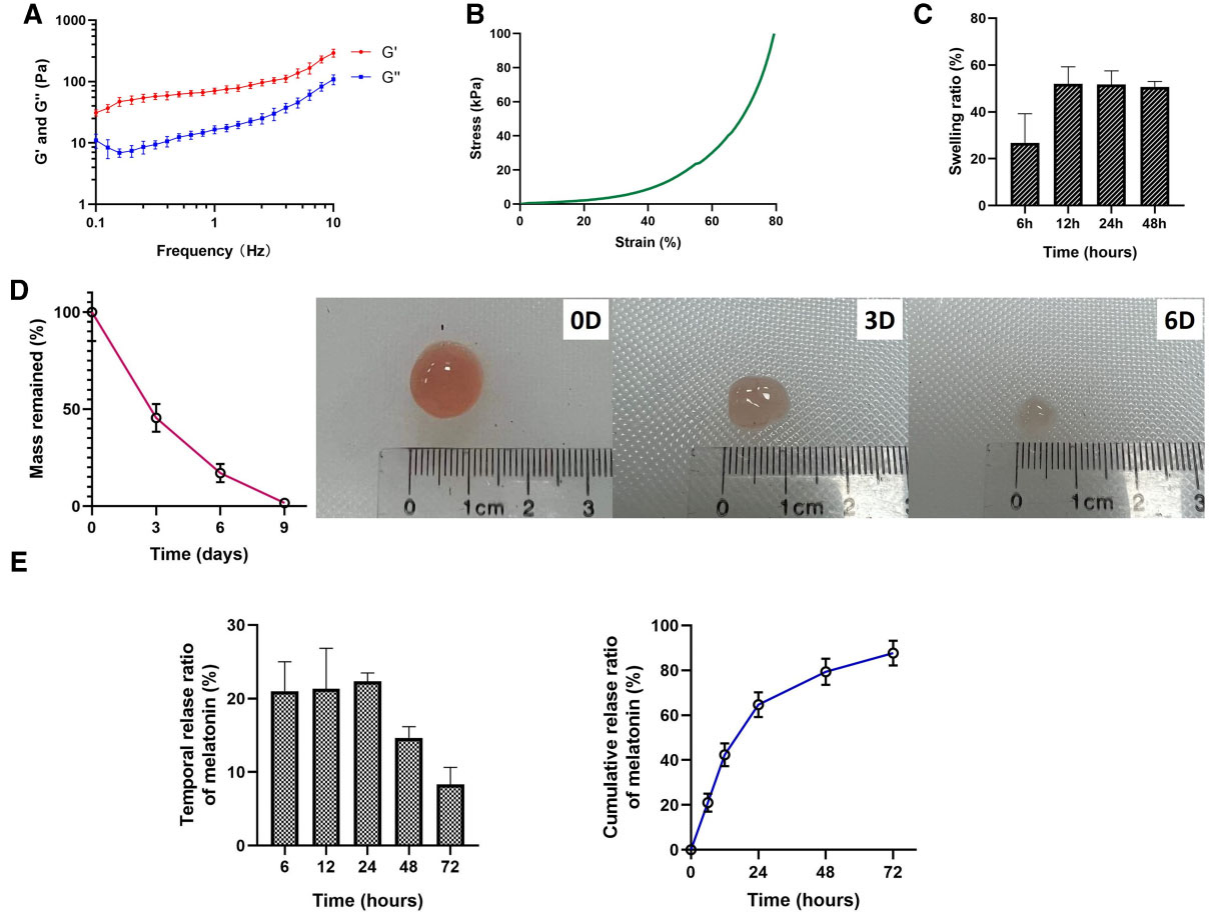
Physical properties of AM gel. (**A**) Rheological analysis of the AM gel. Storage (G′) and loss (G″) moduli as a function of the frequency demonstrated an effective cross-linking of AM gel, *n* = 3. (**B**) Compressive stress–strain characterization of AM gel. (**C**) Swelling ratio from 6 h to 48 h, *n* = 3. (**D**) Mass retention during *in vitro* degradation of AM gel and representative photographs of morphology changes, *n* = 3. (**E**) Temporal and cumulative release of melatonin from AM gel, *n* = 3

Rheological analysis revealed that G′ was over G″ and demonstrated an effective cross-linking of AM gel (Fig. 2A). Compressive stress–strain curves characterized the mechanical properties of AM gel (Fig. 2B), and compressive modulus was 11.24 ± 0.73 kPa. The swelling test indicated that the water sorption capacity of AM gel was low (nearly 50%) and it reached the highest SRs after 12 h (Fig. 2C). For degradation analysis, AM gel was almost completely degraded on Day 9 while incubated in collagenase solution (Fig. 2D). The temporal and cumulative release of melatonin from AM gel was also investigated, revealing that more than 60% melatonin was released within 24 h of gelation and the majority was released after 72 h (Fig. 2E); few melatonin releases were detected after 72 h.

### AM gel significantly facilitated skin wound healing in diabetic rats

**Figure 3.**
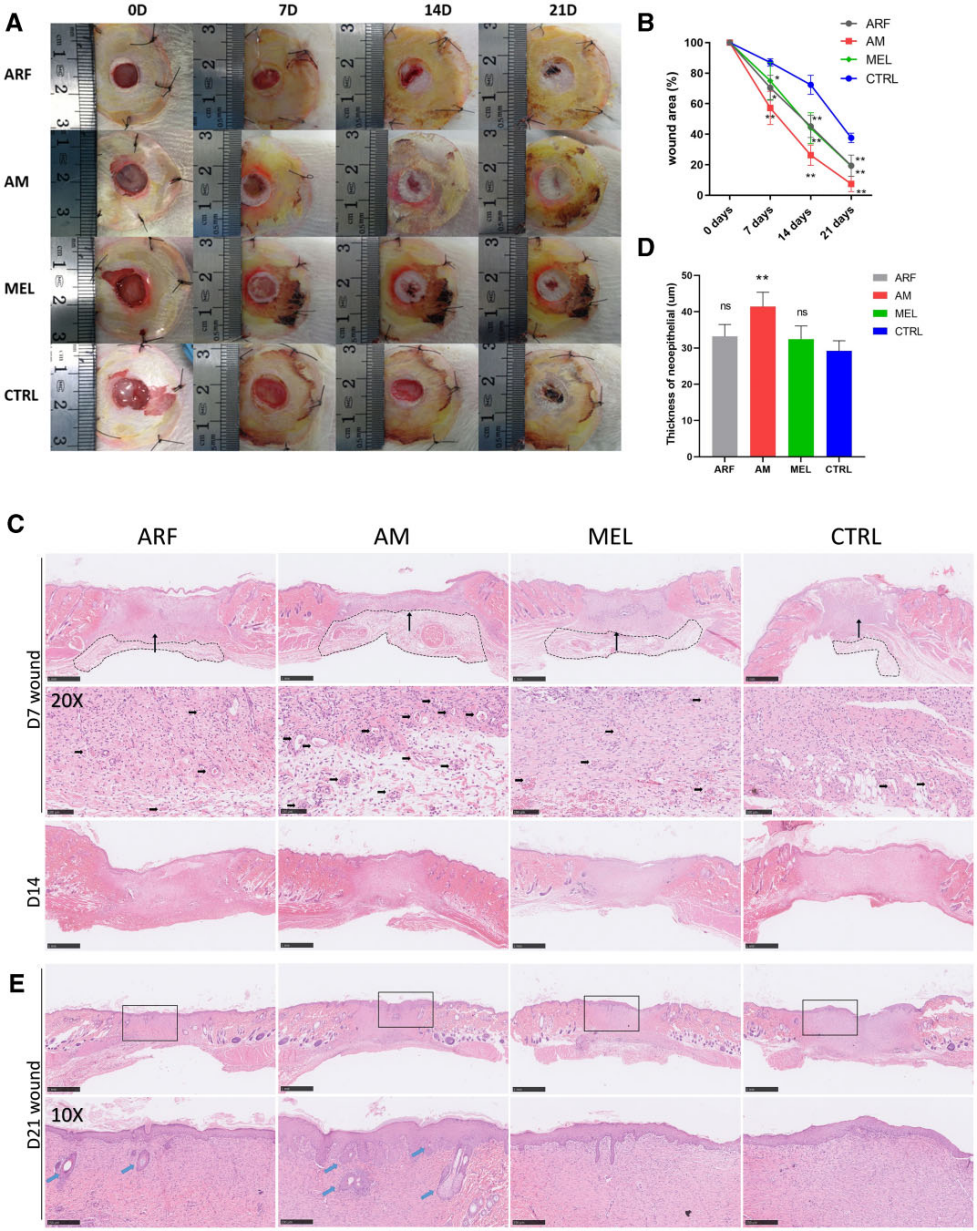
ARF-MEL promotes chronic wound closure in STZ-induced diabetic rats. (**A**) Representative images depicting the process of wound closure on Days 0, 7, 14 and 21. (**B**) Wound closure rates were analyzed at each time point (one-way ANOVA). Data are presented as the means ± SD. *n* = 10. **P < 0.01 compared with the control group. Error bars represent SDs. (**C**) Representative H & E-stained images of wounds on Days 7 and 14, granulation tissue were indicated by the black dotted rectangular, scale bar, 1 mm; higher magnification images (×20) indicate an area denoted with long arrow in the upper image (Day 7), the small arrows point to the newly formed capillaries, scale bar, 100 μm. (**D**) Thickness of neoepithelial in wound edges was calculated in Day 7 (one-way ANOVA), *n* = 5 rats per group. **P < 0.01 compared with the control group. Error bars represent SDs. (**E**) Representative H & E-stained images of wounds on Day 21 and rectangles indicated the higher magnification observation area, scale bar, 1 mm; the arrows point to the hair follicles and sebaceous glands in higher magnification (×10), scale bar, 250 μm

At present, growth factors are widely used to repair wounds [33]. In our study, growth factors were extracted, collected and formulated as an ARF biogel, which is a pharmaceutical delivery system that can be used as a melatonin carrier. We further investigated whether the utilization of ARF and melatonin (AM) can enhance the closure rate in diabetic skin wounds, which are difficult to heal due to persistent inflammation. One week after STZ treatment, wounds were covered with different kinds of biogels (ARF, melatonin, ARF + melatonin) using PBS as the control. As expected, the results revealed that the closure rate of diabetic wounds treated with AM gel was remarkably faster than that of the control group (Fig. 3A). The analysis of the wound areas indicated that AM-treated wound healing was higher in diabetic rats compared with the other groups on Days 7, 14 and 21, and the diabetic wound of AM-treated rats was approximately recovered on Day 21 (Fig. 3B). These results demonstrated that the wound area of the AM group had a higher healing rate than the other groups at each time point. Therefore, wound healing was significantly enhanced in the AM group compared with the other groups. In addition, this result shows that melatonin could promote diabetic wound closure, which could mean that the restoration of posttraumatic immune tissue inflammation patterning, rather than simply applying ARF, played a key role in diabetic wound healing. As illustrated by the H & E staining of the wound section, the granulation tissue thickness of the AM groups was markedly increased on both Days 14 and 21 compared to the other groups. On Day 21, the skin of the subjects in the AM group revealed normal epithelial cells, hair follicles and sebaceous glands; however, the least effects were observed in the control group,. In the higher magnification image, AM groups displayed abundant capillaries in the granulation tissue, whereas less granulation tissue and neovascularization were observed in the control groups by Day 7 after surgery (Fig. 3D). Collectively, the utilization of ARF and melatonin effectively promoted cutaneous wound healing in diabetic rats.

### AM gel promoted vascularization and collagen deposition in the diabetic wound

**Figure 4.**
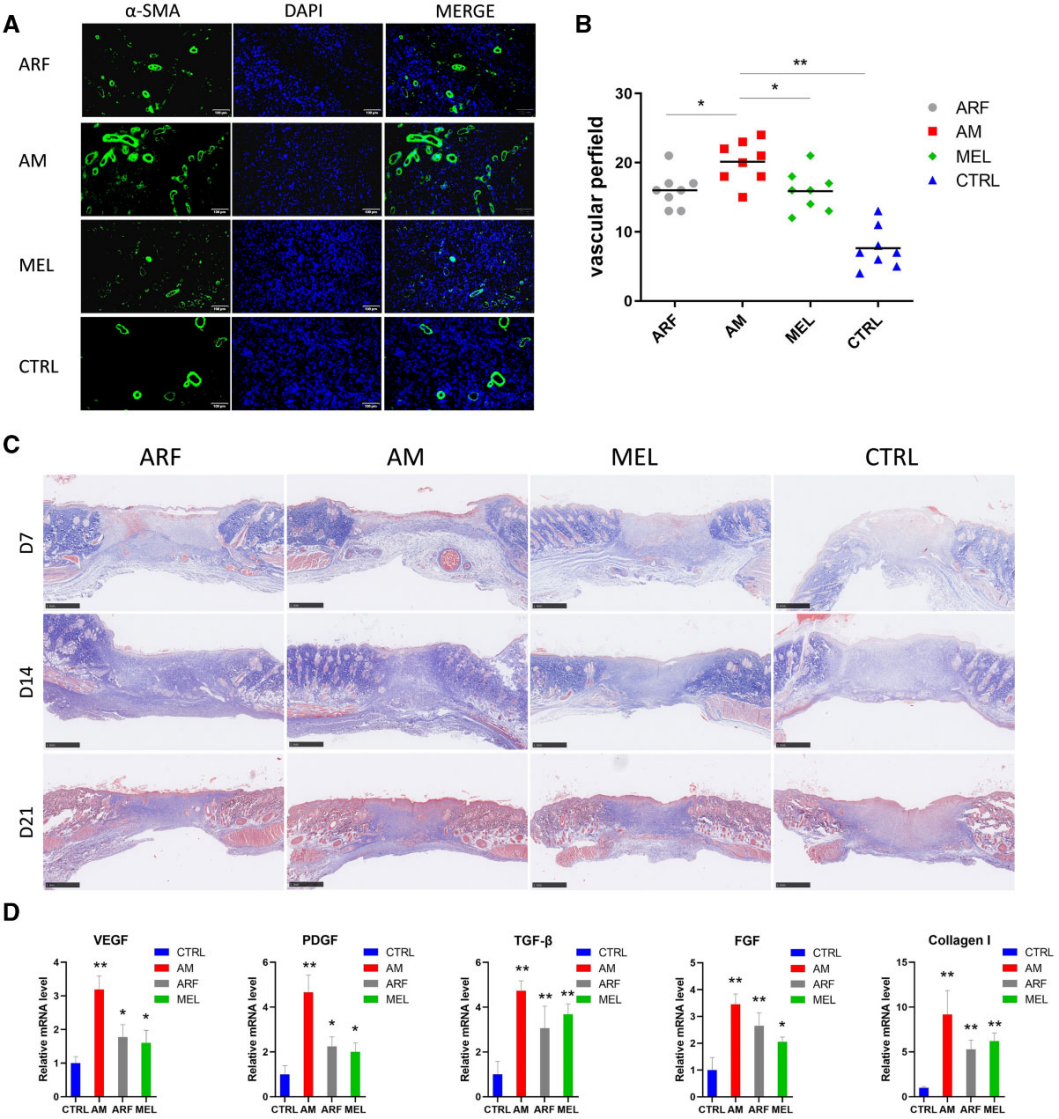
AM gel accelerates angiogenesis and collagen deposition in diabetic wounds. (**A**) New blood vessels were observed on Day 7 by α-SMA staining on enlarged images, scale bar, 100 μm. (**B**) Quantification of the number of new blood vessels (one-way ANOVA). *P < 0.05, **P < 0.01 and comparison with the AM group. *n* = 6–8. (**C**) Masson’s trichrome staining of wound in different treatment groups on Days 7, 14 and 21, scale bar, 1 mm. (**D**) Real-time PCR analysis of VEGF, PDGF, FGF, TGF-β and Collagen I was performed on Day 7 (one-way ANOVA); **P < 0.01 and compared with the control group. *n* = 3. Error bars represent SDs

New tissue formation depends on neovascularization and extracellular matrix production [34]. Vessel regeneration is dispensable for new tissue growth, which is affected by the hyperglycemic microenvironment in diabetic wounds. We used immunofluorescent staining for α-SMA on Day 7 after surgery to mark the formation of new blood vessels (Fig. 4A). As presumed, the AM group exhibited an increased quantity of positive α-SMA staining in the wound sites compared to the other groups, and the control groups showed the least number of capillaries (Fig. 4B). Masson’s trichrome staining revealed that the AM group displayed greater collagen deposition (Fig. 4C). Moreover, we quantified the mRNA levels of angiogenesis (VEGF and PDGF) and fibroblast activation (FGF, TGF-β, Collagen I) in the sample on Day 7 after wounding (Fig. 4D). In accordance with previous findings, the AM group significantly increased compared with the control group. Based on these data, AM gel accelerated vascularization by upregulating VEGF and PDGF levels, and enhanced the formation of granulation tissue by promoting fibroblast proliferation.

### AM gel alleviated inflammation by affecting the proliferation of macrophages

**Figure 5.**
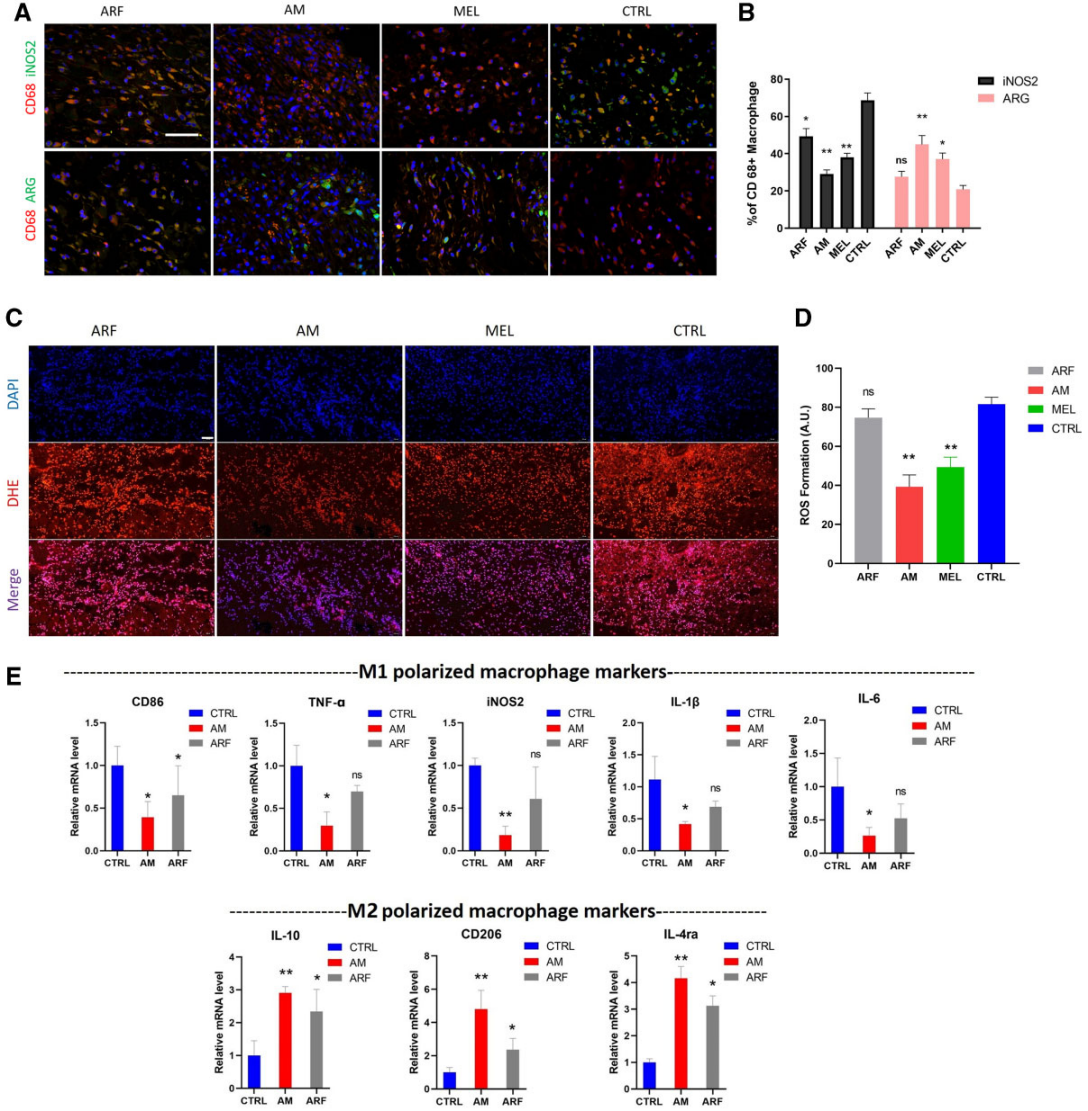
ARF-MEL induces the alternative activation of macrophages in a diabetic wound. (**A**) In wound tissues, dual immunofluorescence staining was performed with CD68 (red) and ARG (green) or iNOS2 (green) on Day 7, scale bar, 20 μm. (**B**) Enumeration of the corresponding cells from the immunofluorescence staining images and the numbers of CD68 +/iNOS2 + (M1 polarized) and CD68 +/CD206 + (M2 polarized) cells per microscopic field. Data are presented as the mean cell numbers ± SD in 5–8 random views per sample from five samples (one-way ANOVA). *P < 0.05 and **P < 0.01 compared with the Ctrl group. *n* = 5–8. Error bars represent SDs. (**C**) The extent of ROS production in wound tissue was determined by dihydroethidium staining on Day 7, scale bar, 100 μm. (**D**) Data are presented as the mean cell numbers ± SD in three random views per sample from three samples (one-way ANOVA). **P < 0.01 compared with the Ctrl group. *n* = 3. Error bars represent SDs. (**E**) The RNA sequencing data (RNA-Seq) for CD86, CD206, IL-4ra, IL-6, IL-10, TNF-α, iNOS2 and IL-1β were verified using real-time PCR on Day 7 (one-way ANOVA). *P < 0.05 and **P < 0.01 compared with the Ctrl group. *n* = 3. Error bars represent SDs

Immunofluorescence staining was performed on Day 7 post-surgery. The expression of the M1 polarized macrophage marker iNOS2 was downregulated, whereas that of the M2 polarized macrophage marker ARG was upregulated in the AM group (Fig. 5A and B). The formation of ROS was significantly decreased in the AM and MEL group (Fig. 5C and D), suggesting that AM gel and melatonin could inhibit posttraumatic oxidative stress. Further quantification of the mRNA levels in the tissue revealed that several inflammatory cytokines, including IL-1β, CD86, TNF-α and IL-6, were markedly decreased in the AM groups, while several M2 polarized macrophage markers, including CD206, IL-4ra and IL-10, were upregulated, indicating that the AM induced M2 macrophage polarization and restrained the inflammatory reactions during the healing period (Fig. 5E). Therefore, it is evident that the combined utilization of ARF and melatonin facilitated the polarization of macrophages toward reducing ROS formation and inflammatory responses.

### AM gel affects the inflammatory response during diabetic wound healing

**Figure 6.**
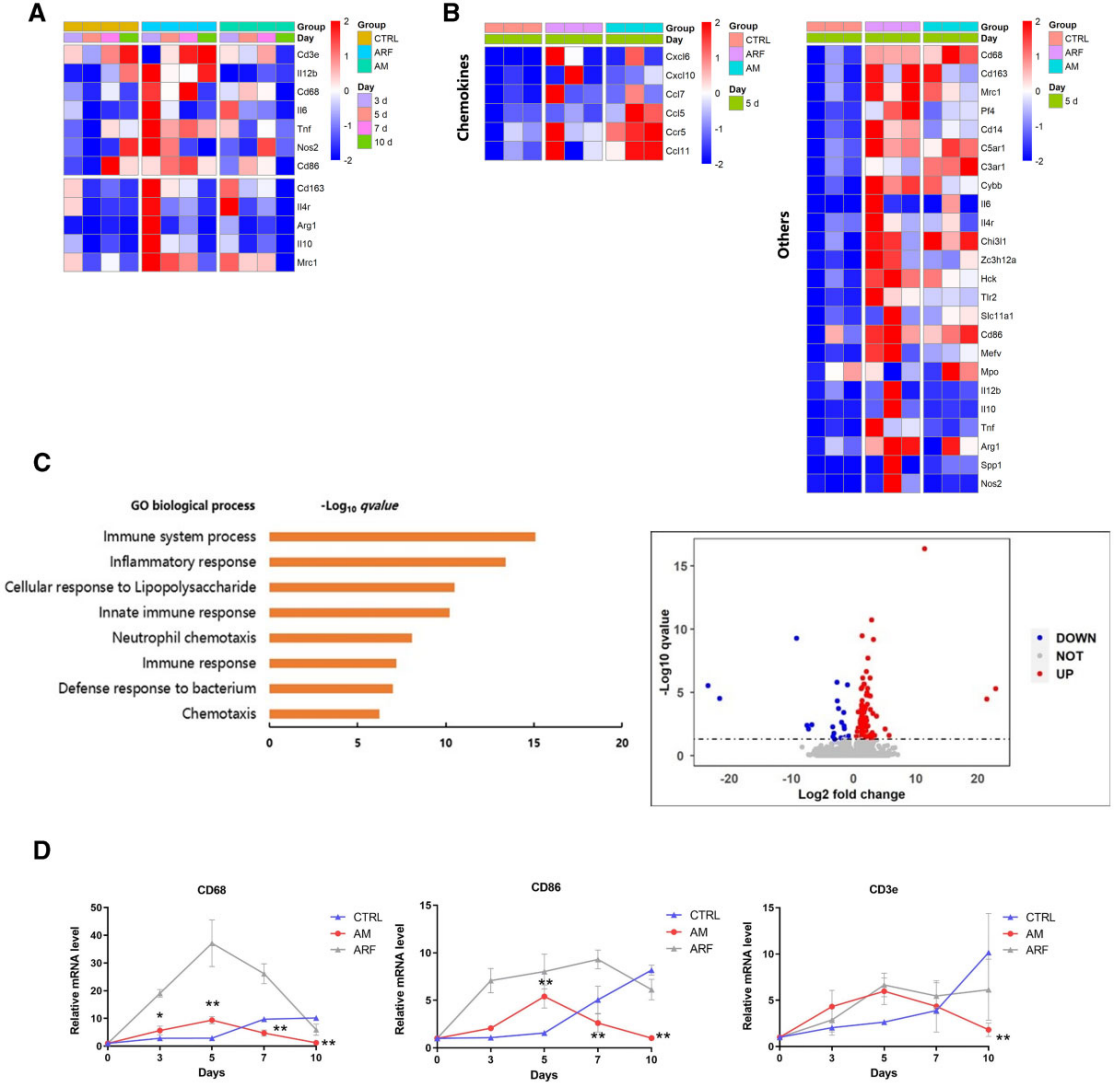
The effect of AM gel on the inflammation of diabetic wounds. (**A**) Heat map of the RNA-Seq analysis result shows relative expression of immune-related gene transcripts, and reveals differentially expressed immune-related transcripts in AM-treated, ARF-treated and control groups. (**B**) Relative mRNA expression of chemokines and other inflammatory regulators, on Day 5. Three fields for each gene from each group represent technical replicates. (**C**) Volcano plots indicate differences in expression of genes between the AM-treated and control groups, and the enriched biological process GO analysis. (**D**) Real-time PCR was used to evaluate the quantification of the immune cell mRNA levels during wound healing (Days 0, 3, 5, 7 and 10, one-way ANOVA). Data are presented as mean ± SD. *n* = 3. *P < 0.05, **P < 0.01 compared with the control group. Error bars represent standard deviations (SDs)

The gene expression profile was analyzed using RNA-Seq. The data revealed that pro-inflammatory genes (*CD3e, CD86, Nos2, TNF, IL-12b*) were less active and upregulated continuously throughout the healing process in the diabetic cutaneous microenvironment without intervention. The expression of genes involved in inflammatory responses was maintained on Day 10. Moreover, anti-inflammatory M2-like phenotype-related genes (*CD163, ARG1, IL-10, IL-4r, Mrc-1*) were markedly downregulated after Day 3 (Fig. 6A). In contrast, the AM and ARF groups revealed upregulated expression of genes involving inflammatory responses from Days 0 to 5 (Fig. 6A), respectively, suggesting both AM and ARF groups were switching from chronic to acute inflammatory response during the early wound healing process when compared to the control group; however, on Day 7, inflammation-related genes (*CD3e, CD86, CD68, Nos2, TNF, IL-12b*) still maintained a high expression level in the ARF group, and whereas inflammation-related genes began to decrease in the AM group. On Day 10, inflammation-related genes in the ARF group were tending downwards, and the AM group had completely decreased while the control group was still increasing. We evaluated the gene expression profile by RNA-Seq on Day 5 as a consequence. This result indicated a significant difference in the expression of genes involved in chemokine-mediated immune responses and phenotypic markers (Fig. 6B). To assess the possibility of AM gel regulating diabetic wound inflammation, we analyzed the significant differentially expressed gene between the AM gel treatment (AM group) and the control group using RNA-Seq and identified 134 genes (q < 0.05, fold change > 2) on Day 5. The majority of these genes were positively correlated with genes controlling the immune system process and inflammatory response (Fig. 6C). Furthermore, we evaluated the quantification of the mRNA levels using real-time polymerase chain reaction and showed an acute inflammation response in the AM gel-intervened microenvironment, which was upregulated remarkably from Days 0 to 5 and reverted to baseline by Day 10 (Fig. 6D).

### ARF-MEL combined effect decreases AGE-induced apoptosis and improves the function in HUVEC

**Figure 7.**
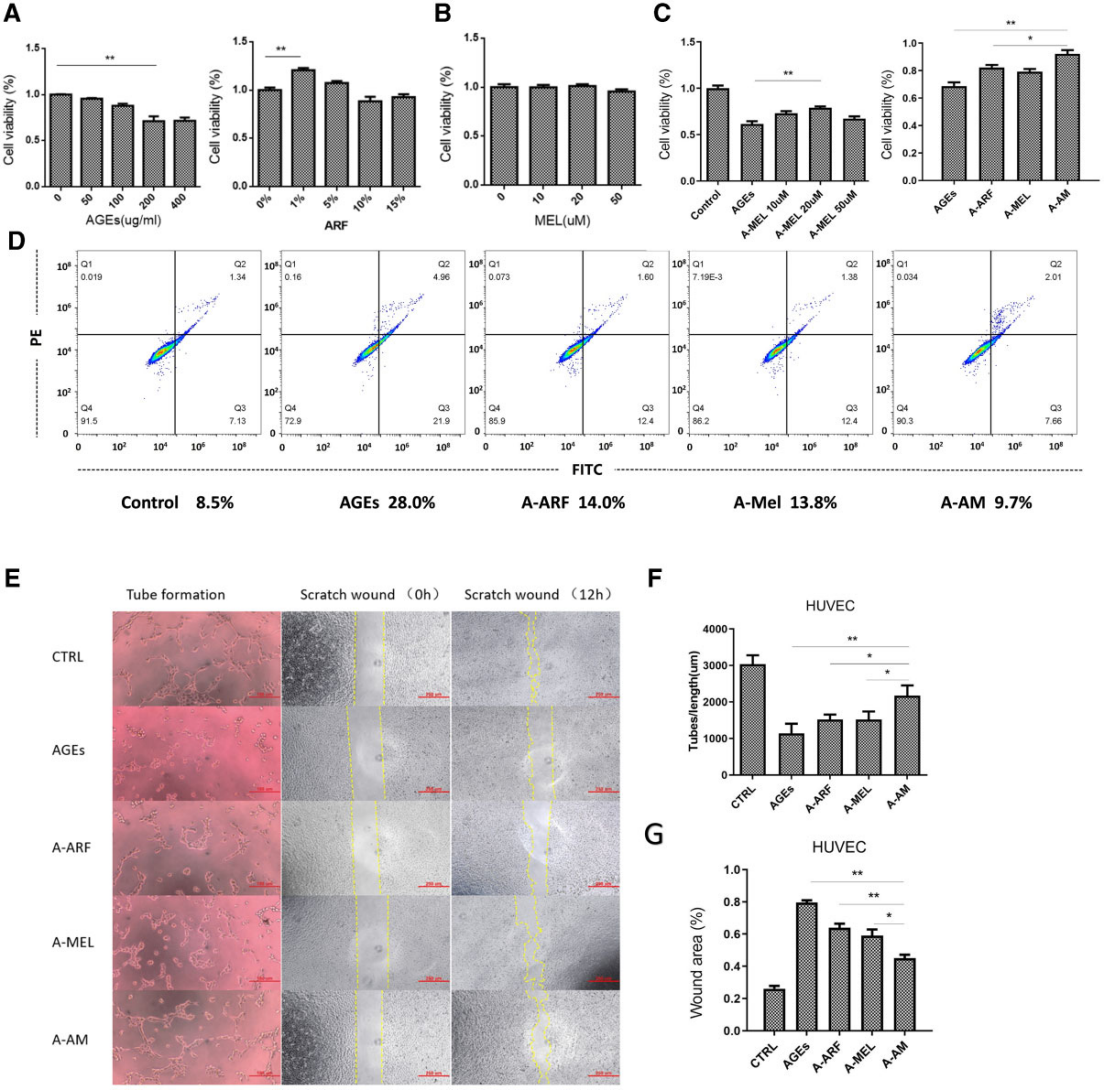
Treatment with ARF and melatonin decreases AGE-induced apoptosis in HUVEC. (**A**) The results of the cell counting kit-8 on HUVEC that received different concentrations of AGEs and ARF for 24 h (one-way ANOVA). Error bars represent SDs. (**B**) Proliferation rates of melatonin-treated HUVEC were assessed by using the CCK-8 kit (one-way ANOVA). Error bars represent SDs. (**C**) The proliferation rate of HUVEC cultured in an AGE environment and treated with ARF or different concentrations of melatonin was analyzed using the CCK-8 kit (one-way ANOVA). *P < 0.05, **P < 0.01. *n* = 5. Error bars represent SDs. (**D**) The apoptosis rate (Q2 + Q3) of HUVEC, treated with ARF and melatonin in AGEs was detected using the annexin V-FITC/PI kit. (**E**) Tube formation assays and scratch healing assays were performed on HUVEC treated with ARF and melatonin in AGEs for 12 h, scale bar of tube formation image, 100 μm; scale bar of scratch healing assays image, 250 μm. (**F** and **G**) The tube length and scratch area were quantified via image analysis (one-way ANOVA). Significant differences between the treatment and control groups, as indicated as *P < 0.05, **P < 0.01 compared with the A-AM group. *n* = 3. Error bars represent SDs

The cell viability of HUVEC decreased in the AGE microenvironment in a dose-dependent manner, and the reduced percentage of cells reached a peak at a concentration of 200 μg/ml. The CCK-8 assay demonstrated that ARF was able to promote HUVEC proliferation at concentrations ≤ 5% after 24 h of treatment and maximized at 1% (Fig. 7A). Melatonin was not cytotoxic and did not facilitate the proliferation of HUVEC at concentrations of ≤ 50 μM after 24 h (Fig. 7B); however, in the AGE microenvironment, melatonin had a greater protective effect against cell apoptosis at a concentration of 20 μM (Fig. 7C). We analyzed the combined effect of ARF and melatonin in the AGE microenvironment, and the results revealed a remarkable attenuation of HUVEC apoptosis in the combined treatment (Fig. 7C). However, the AGE microenvironment did not inhibit the proliferation of keratinocytes (Hacat, Fig. S2A). Apoptosis was detected using the annexin V-FITC/PI kit; and the result showed that the percentage of apoptosis decreased in the AM treatment group (Fig. 7D). Collectively, ARF and melatonin in combination were more efficient in attenuating AGE-induced apoptosis in HUVEC than when they were used alone.

We evaluated the efficacy of AM treatment on neovascularization and migration in AGE-treated HUVEC using tube formation and scratch wound assays. Representative micrographs of HUVEC in each treatment group revealed capillary-like structures and cell migration (Fig. 7E). First, the group that received AM treatment displayed increased total length of neovascularization compared with the groups treated with ARF or melatonin, and greatly increased angiogenesis compared with the AGE groups (Fig. 7F). Second, compared to the AGE-treated group, AM effectively protected the migration ability of HUVEC with markedly decreased artificial wound areas, and the ARF and melatonin groups revealed slightly improved cell migration in the AGE microenvironment (Fig. 7G). Collectively, the combined use of ARF and melatonin can be highly efficient in recovering the cell function that is weakened by AGEs.

### ARF–MEL affected the paracrine AGE-induced macrophages and facilitated cell activation

**Figure 8.**
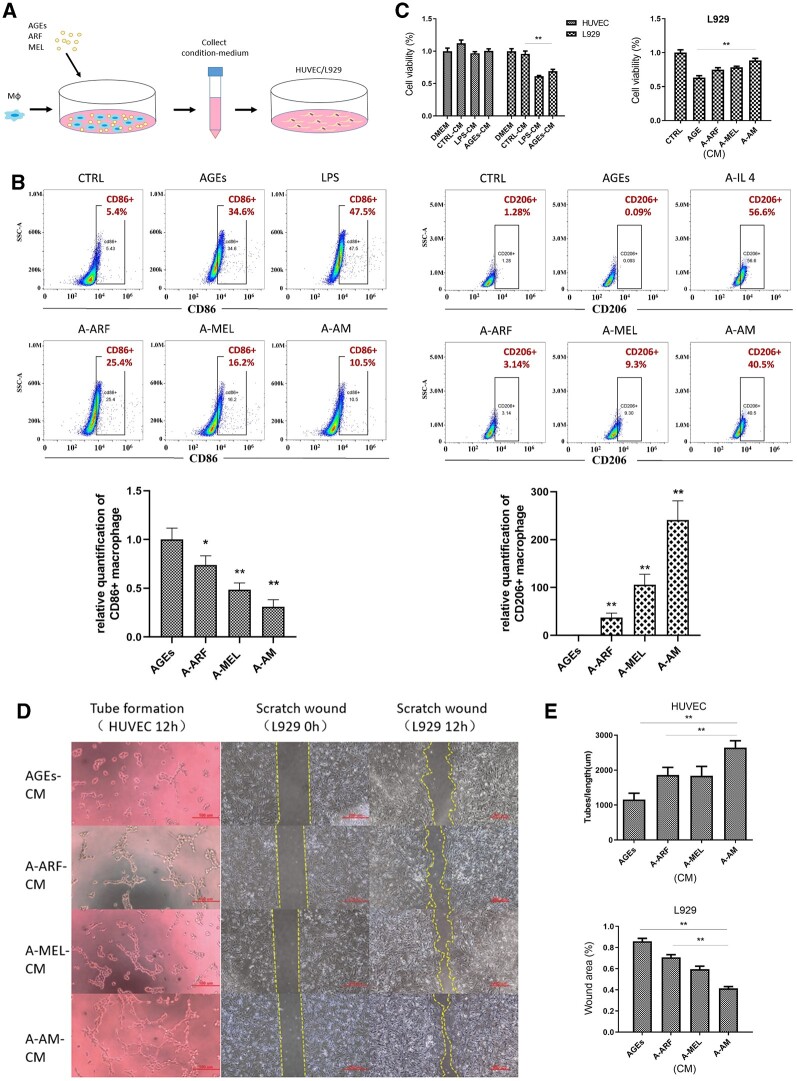
The proliferation, migration or tube formation of L929 and HUVEC are produced by culture medium (CM) from AGEs-ARF-MEL-induced macrophages, AGEs-induced macrophages, and unstimulated macrophages. (**A**) Schematic depicting the process of paracrine signaling from the AGE-ARF-MEL-treated macrophage conditioned medium. (**B**) Representative flow cytometry results of the CD86+ and CD206+ populations in macrophages treated with 100 ng/ml LPS (M1 polarized), 40 ng/ml IL-4 (M2 polarized), AGEs (100 μg/ml), ARF 1% and MEL 20 μM compared with the untreated control. *P < 0.05 and **P < 0.01 compared with the control group. *n* = 5–8. Error bars represent SDs. (**C**) The proliferation rate of HUVEC and L929 cells pretreated with different CMs was determined using the CCK-8 kit (one-way ANOVA). **P < 0.01 compared with the control-CM group or AGEs group. N = 5. Error bars represent SDs. (**D**) A tube formation assay of HUVEC and scratch healing assay of L929 were treated with different CMs for 12 h, scale bar of tube formation image, 100 μm; scale bar of scratch healing assays image, 250 μm. (**E**) The tube length and scratch area were quantified using image analysis (one-way ANOVA). **P < 0.01 compared with the A-AM group. *n* = 3. Error bars represent SDs

AM treatment effectively promoted vascularization and collagen deposition in diabetic wounds. The paracrine signaling of M1-polarized macrophages is one of the causes of vascular endothelial cell and fibroblast dysfunction. In particular, IL-1β, TNF-α and IL-6 and such inflammatory factors limit cell viability, which is considered to be the result of paracrine signaling from M1-polarized macrophages. Furthermore, the hyperglycemic microenvironment reduces macrophages to M2 type polarization, which is anti-inflammatory and promotes fibroblast proliferation and migration. To verify this hypothesis, we used a CM to treat HUVEC and L929 cells which were collected from AM-stimulated macrophages for 48 h (Fig. 8A). Flow cytometry was used to evaluate the macrophage polarization status of RAW 264.7 cells after treatment with ARF and melatonin in the AGE microenvironment for 48 h. The percentage of CD86 + macrophages treated with AGEs was similar to that of LPS-stimulated macrophages. When treated with ARF and melatonin following the administration of AGEs, the positive rate of CD86 was markedly decreased (Fig. 8B, Supplementary Fig. S3), indicating that AM reduced macrophage polarization to the M1 type. The percentages of CD206 + macrophages in AGEs treated with AM were increased compared to the treatment of the ARF, melatonin, or untreated groups (Fig. 8B, Supplementary Fig. S3), and this result indicated that AM has the potential to induce M2 type polarization in macrophages.

To evaluate the effect of paracrine signaling, CM from macrophages or AGE-induced macrophages was added to HUVEC and L929 cells. It has already been confirmed that LPS induces macrophage to M1 type polarization and causes inflammation. We also collected the CM of LPS-induced macrophages for comparison. According to the CCK-8 results, L929 cells exposed to AGE-CM or LPS-CM revealed remarkably lower proliferation rates than the other two groups; however, unexpectedly, the proliferation rates of HUVEC exposed to AGE-CM were not significantly different from those of the other groups and persistent inflammation did not inhibit the proliferation of endothelial cells (Fig. 8C). We also tested the anti-inflammatory effect of AM treatment in AGEs (A-AM), and found that the proliferation of L929 exposed to A-AM-CM (condition medium of AM-treated macrophages in AGEs) was markedly higher than in other AGE-containing groups and similar to common medium that was AGEs-free (Fig. 8C); similar results were demonstrated in the testing for keratinocytes (Hacat, Supplementary Fig. S2B). After adding the CM to epithelial cells (HUVEC) or fibroblasts (L929), the group that received A-AM-CM displayed increased angiogenesis (HUVEC) and migration (L929), compared to any other group (Fig. 8D and E).

## Discussion

To promote the healing of diabetic wounds, we need to understand the wound repair mechanisms to improve the treatment regimens, particularly the inflammatory responses that affect tissue damage, repair and regeneration in the tissue microenvironment [35]. In diabetic skin wound healing, the posttraumatic homeostasis and inflammatory state are characterized by hyperglycemia-induced persistent chronic inflammation and high levels of inflammatory cytokines [36], which often leads to retarded tissue regeneration [37]. Moreover, we found that the posttraumatic microenvironment in the diabetic wound was in a state of low-level inflammation that gradually increased, which was characteristic of the compromised microenvironment homeostasis.

**Figure 9.**
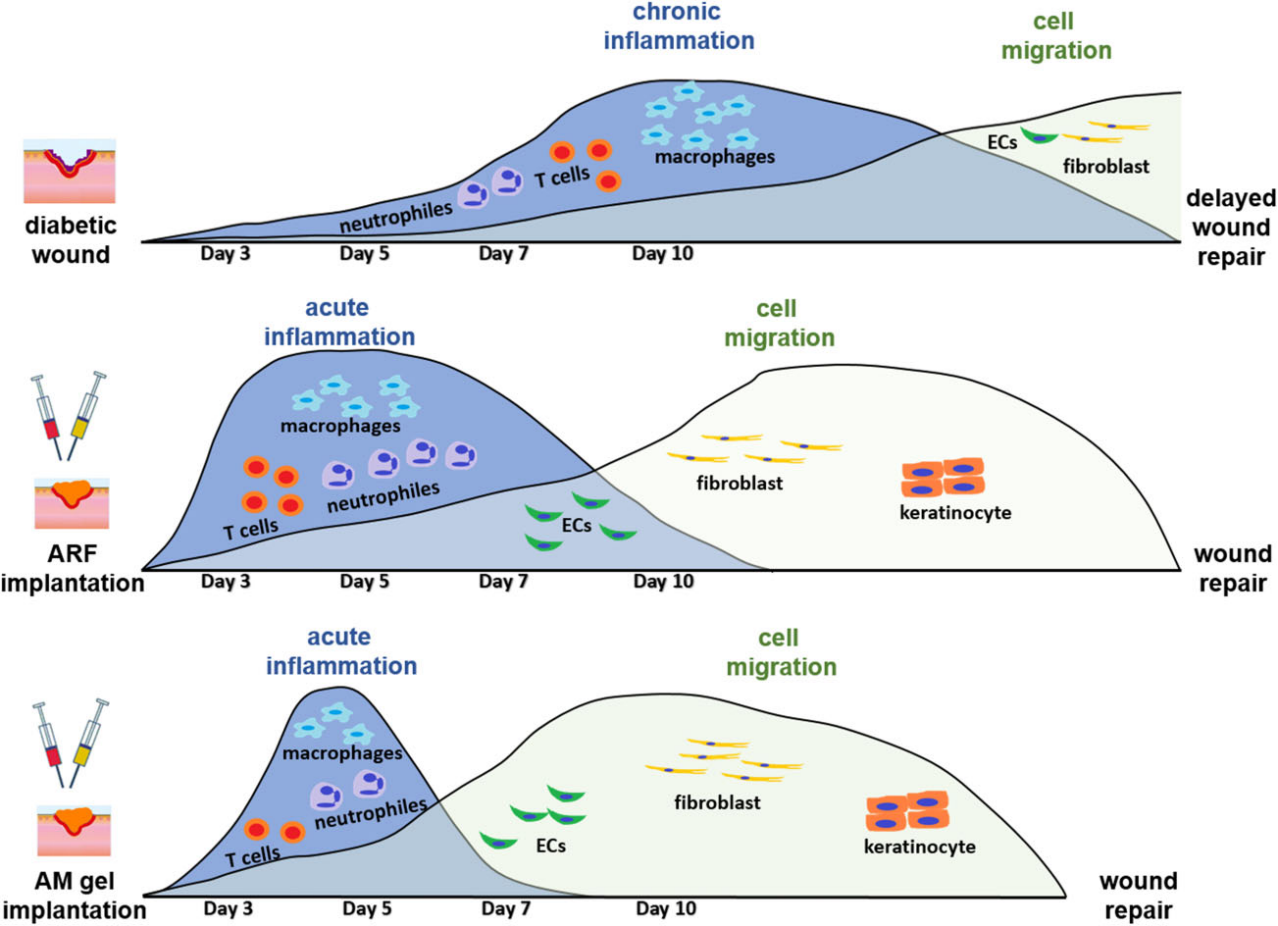
Schematic of accurate and proactive modulation via ARF and AM gel altering ‘inflammation patterning’ and improving tissue regeneration. ECs, endothelial cells

Our study confirms our hypothesis that there is a (Fig. 9) compromised microenvironmental homeostasis in diabetic wounds, and the remodeling of this patterning has a remarkable effect on homeostatic restoration and the healing process. The inflammatory patterning in diabetic wounds shows that inflammatory factors proliferate tardily and continuously, and lead to postponed repair and regeneration, which is characterized by hindering the migration of vascular endothelial cells, fibroblasts, and keratinocytes. Meanwhile, the acute-like inflammation caused by ARF in diabetic cutaneous defects is unregulated, which is distinguished from the inherent healing microenvironment of non-diabetic wounds. If this excessive inflammation is not controlled, the prolonged acute inflammation can cause lasting indirect damage to functional cells within the microenvironment, thus leading to retarded tissue regeneration [38, 39]. Suitable pro-inflammatory responses have been identified as modes of optimal wound regeneration [40] rather than the acute and excessive inflammatory microenvironment that results from ARF induction. After treatment with AM gel, the tissue microenvironment can be immediately altered into an acute inflammatory state; however, in the next stage, the pro-inflammatory factors rapidly decrease and anti-inflammatory factors are retained (Fig. 9). The particular inflammatory patterning that mimics the accurate and proactive immunomodulation of the posttraumatic state caused by AM gel is found to be more suitable for diabetic skin tissue reconstruction. Active immune regulation can restore homeostasis of the posttraumatic cell microenvironment in diabetic wounds, thereby reviving the activity of cytokines and growth factors and promoting cell proliferation and differentiation.

Recent research has revealed that therapeutic approaches that can activate acute wound healing responses are highly effective in promoting wound healing [40]. The early onset acute inflammatory phase is an important stage for successful wound closure, and we demonstrated that ARF is capable of this similar function; however, the posttraumatic acute inflammatory phase caused by ARF is difficult to control or regulate, and diabetic microenvironmental homeostasis has not been effectively restored, eventually developing into overexpression and leading to disordered tissue regeneration. Hence, the design of innovative AM gel is based on restoring the ordered inflammatory stages, thereby altering prolonged chronic inflammation, and alleviating excessive acute inflammation. Recent attempts to suppress the overexpressed inflammatory phase have focused on the effects of melatonin [41]. AM gel is an innovative treatment that contains autologous regeneration growth factors and melatonin. The results of RNA-Seq revealed that both AM and ARF could trigger an acute inflammatory state in the initial stage, which is a suitable start-up for wound healing. Nevertheless, as healing progresses, prolonged and excessive acute inflammation triggered by ARF does not confer benefits in the middle and late stages of healing. Blindly provoking M1 polarized macrophage differentiation by excessive acute inflammation without considering the timeline is definitely harmful to prognosis [42]. In contrast, the posttraumatic inflammatory patterning re-established by AM gel demonstrated superior tissue regeneration in diabetic rats. The addition of melatonin removed the disordered inflammation in the tissue microenvironment caused by ARF. Meanwhile, melatonin-enriched ARF biogel increased the expression of anti-inflammatory mediators (including IL-10 and IL-4), suggesting that melatonin inhibits excessive acute inflammation through its anti-inflammatory effects. This therapy, based on the use of ARFs in combination with antioxidants, provides an alternative approach to wound healing that involves accurate and proactive immunomodulation to restore microenvironment homeostasis and addresses the potential for persistent inflammation, and dysfunction of growth factors.

Indeed, the change in inflammatory patterning is influenced by the proliferation and polarization of macrophages. The polarization of macrophages has gained remarkable attention because macrophage polarization is crucial in inflammatory and immune processes. The interaction between macrophages and other immune cells determines the outcome of angiogenesis [43]. Wound healing results from the complicated communication network between epithelial and immune cells [44]. The macrophage phenotype between the two extremes, ranging from pro-inflammatory (M1 polarized) to anti-inflammatory (M2 polarized) phenotypes, greatly influences wound healing [45] and biomaterial performance [46]. During wound healing, different phenotypes of macrophages change over time, which is crucial for the removal of debris, migration of cells [47], induction of angiogenesis, collagen secretion [48] and extracellular matrix synthesis [49]. Designed to accelerate diabetic skin wound healing by aiming at macrophages by focusing on regulating or transforming the phenotypes of macrophages is a feasible strategy [50]. Based on our current findings, AM gel has specific anti-inflammatory properties after proactive-induced acute inflammation because it stimulates macrophages, reduces M1 polarized phenotypes and promotes M2 polarized phenotypes in diabetic skin wounds. This can be considered a metaphase representation of the inflammatory patterning affected by AM gel, and the results are consistent with our observations of the wound tissue status on Day 7. Several factors that enhance M1-polarized macrophages, such as TNF-α, iNOS and IL-6, have been verified to exert an intense pro-inflammatory effect [51]. Similarly, the factors that promote M2 polarized macrophages, such as IL-4 and IL-10, have been demonstrated to produce the intensity of anti-inflammatory effects [52]. The variation trend of these factors is consistent with our results from the M1/M2 polarized ratio and diabetic skin wounds, demonstrating that AM gel-treated wounds exhibited a significant anti-inflammatory effect at Day 7. It must be noted that the limitations of the M1/M2 polarized model for defining macrophage polarization and the lack of a standard to define macrophage activation in experiments impede progress in multiple ways [53]. There is still no unanimous acceptance of a standardized nomenclature in the scientific community.

In diabetic cutaneous defects, the accumulation of AGEs in the hyperglycemic microenvironment also impacts extracellular and intracellular structure and function [54], leading to the inhibition of angiogenesis during wound repair. Our results suggest that AM gel mitigates the effects of AGEs, directly stimulates the differentiation of macrophages, improves the cellular tissue microenvironment and increases angiogenesis and cell activity through paracrine signaling from reparative macrophages. Tissue regeneration occurs through a series of processes, including the proliferation, migration and maturation of cells [55]. Collative evidence indicates that the accumulation of AGEs is another mechanism that causes tissue damage in diabetes patients. It has been reported that AGEs are a crucial factor in the impairment of cell activity, inflammatory induction and pathogenesis of diabetic skin tissue [56]. In our research, AGEs led to L929 dysfunction and resulted in apoptosis. Moreover, the functions of cells (HUVEC or L929), including tube formation and migration, were impaired after either AGE or AGE-induced macrophage condition medium treatment. Macrophages gradually become pro-inflammatory under the influence of AGEs. After treatment with a combination of ARF and melatonin, the functions of the cells were restored and apoptosis was reduced in the AGE-treated microenvironment. This may be attributed to ARF reducing the expression of apoptosis-related genes [57], and melatonin protecting cells against apoptosis and dysfunction via autophagy flux stimulation [58]. The phenotype of AGE-induced macrophages transforms from pro-inflammatory to anti-inflammatory after ARF-melatonin cocktail treatment. Traditionally, regenerated skin in diabetic patients is predominantly scar tissue; our results reveal that the immune modulation by AM gel triggered the full regeneration of wound as observed from skin appendages. The reduced inflammation prevented scar formation and was beneficial for full regeneration [59]. Anti-inflammatory macrophages play vital roles in reducing the function of other pro-inflammatory and pro-fibrotic immune cells, thus minimizing inflammation and fibrosis [60]. AM gel has been proven to be capable of remodeling the heterogeneity of immune cells in this research.

In our research, we successfully extracted the ARF, transformed its physical properties and mixed it with an antioxidant (melatonin), eventually producing a freeze-dried powder, which is convenient for clinical application. This kind of compound preparation can be re-dissolved and formed into hydrogels (AM gel) during application, and the special physical condition of AM gel leads to widespread clinical utility and applicability. Platelet concentrates have been clinically used for treating diabetic wounds; however, excessive pro-inflammatory agents such as IL-6, TNF-α and so on [61] within the autologous blood extracts from diabetic patients should be considered. Pro-inflammatory agents are crucial in the pathogenesis of diabetic tissues and the process of diabetic wound healing [62]. The addition of melatonin to form AM gel is designed to minimize the adverse effects of pro-inflammatory agents. Recent research indicated that melatonin effectively alleviates inflammatory responses by switching the macrophage phenotype from M1 to M2 via activation of STAT-3 signaling [18], therefore enhancing the efficiency of ARFs in AM gel and promoting cell proliferation in diabetic environments. Alternatively, allogeneic blood extracts from a non-diabetic healthy donor can be used, especially in severe diabetic patients where the activity of ARF may be highly compromised by pro-inflammatory agents. In such a clinical application scenario, the immunocompatibility and safety of the allogeneic blood extract should be carefully evaluated and considered, although its immunogenicity might not be an obstacle to its clinical application because of the low immunogenicity of platelet [63]. In conclusion, AM gel treatment represents a potential therapeutic approach that remodels posttraumatic inflammation patterning, restores compromising microenvironment homeostasis and improves tissue regeneration. Additionally, experiments *in vivo* demonstrate that AM gel accelerates diabetic cutaneous wound healing. Although several studies have been conducted on diabetic refractory wounds, most of the results cannot be achieved in clinical conditions. AM gel is more likely to be used in the clinic, and this accurate and proactive immunomodulation strategy holds promising prospects for clinical treatment.

## Conclusion

AM gel is more suitable for tissue regeneration due to the specific inflammatory change patterning induced by immune regulation in diabetic wounds. This accurate and proactive immunomodulation can re-establish the homeostasis of the cell microenvironment, regulate the polarization trend of macrophages and thus restore the activity of cell growth factors, promote cell proliferation and differentiation in posttraumatic diabetic wounds, and accelerate wound healing.

## Supplementary data

Supplementary data are available at *REGBIO* online.

## Author contributions

Y.Y., L.W., Y.Z. and Z.-Y.Z. conceived this research and designed experiments; Y.H., S.L., Y.Z., Y.Z., Z.H. and W.L. participated in the design and interpretation of the data; Y.Y. performed experiments and analysis; Y.Y., Q.Z., L.C., Z.L., and W.W. wrote the paper and participated in the revisions of it. All authors read and approved the final manuscript.

## Institutional review board statement

All procedures involved in the manuscript entitled ‘Antioxidant-enriched autologous biogel promoted diabetic wound healing by the regulation of internal environment inflammatory patterning’ were obtained with informed consent and approved by the Ethical Committee of The Third Affiliated Hospital of Guangzhou Medical University. Registration number: 2020-015.

All procedures performed in studies involving human participants were in accordance with the ethical standards of The Third Affiliated Hospital of Guangzhou Medical University and with the 1964 Helsinki Declaration.

All animal experiments were performed following the ethical standard and approved by the Animal Ethics Committee of Guangdong Medical Laboratory Animal Center. Registration number: B202008-5.

## Supplementary Material

rbac023_Supplementary_Data
