# Supplementary material for: Antioxidant-enriched autologous biogel promoted diabetic wound healing by remodeling inherent posttraumatic inflammatory patterning and restoring compromised microenvironment homeostasis
Source: Regen Biomater. 2022 Apr 29;9:rbac023. doi: 10.1093/rb/rbac023 (PMC9071060; doi:10.1093/rb/rbac023)
Supplement: rbac023_Supplementary_Data [file rbac023_supplementary_data.docx]

**Antioxidant-enriched** **autologous biogel promoted diabetic wound healing by remodeling inherent posttraumatic inflammatory patterning and restoring compromised microenvironment homeostasis**

Yixi Yang^*,1,2^, Le Wang^*,1,2,3^, Yonglin Zhou^*,1^, Yijun He^1^, Shaozhang Lin^1^, Yuwei Zeng^1^, Yunhe Zhou^1^, Wei Li^4^, Zaopeng He^4^, Qi Zhao^1^, Lihao Chen^1^, Zijie Li^1^, Wenhao Wang^#,1^, Zhi-Yong Zhang^#,1^

^*^: these authors contributed equally to this work.

^#^: co-corresponding authors.

1. Translational Research Centre of Regenerative Medicine and 3D Printing of Guangzhou Medical University, Guangdong Province Engineering Research Center for Biomedical Engineering, State Key Laboratory of Respiratory Disease, The Third Affiliated Hospital of Guangzhou Medical University, Guangzhou 510150, P. R. China
2. Department of Orthopaedic Surgery, The Third Affiliated Hospital of Guangzhou Medical University, Guangzhou 510150, P. R. China
3. Medical Technology and Related Equipment Laboratory, The Third Affiliated Hospital of Guangzhou Medical University, Guangzhou 510150, P. R. China
4. Hand and Foot Surgery & Plastic Surgery, Affiliated Shunde Hospital of Guangzhou Medical University, Shunde District, Foshan, P. R. China

**Supplementary Data**

|  | Gene | Primer sequence（5’-3’） | | Amplification length（bp） |
| --- | --- | --- | --- | --- |
| 1 | r-GAPDH | Forward： | GCAAGGATACTGAGAGCAAGAG | 98 |
|  |  | Reverse： | GGATGGAATTGTGAGGGAGATG |  |
| 2 | r-VEGF | Forward： | GAAGACACAGTGGTGGAAGAAG | 111 |
|  |  | Reverse： | ACAAGGTCCTCCTGAGCTATAC |  |
| 3 | r-PDGF | Forward： | CCCTAGAGAGCAGAGTGTAAGT | 94 |
|  |  | Reverse： | GTTCTTAGGGCCTTGGATTAGG |  |
| 4 | r-FGF | Forward： | CTCACCTCTGTACCTGGAAATC | 99 |
|  |  | Reverse： | GTGCCGCTCTTCATCTTGTA |  |
| 5 | r-TGF-β | Forward： | GCAACAATTCCTGGCGTTAC | 120 |
|  |  | Reverse： | GTATTCCGTCTCCTTGGTTCAG |  |
| 6 | r-collagen I | Forward： | CCAATGGTGCTCCTGGTATT | 111 |
|  |  | Reverse： | GTTCACCACTGTTGCCTTTG |  |
| 7 | r-MPO | Forward： | TGGCTACCCTACTTCCCATAA | 121 |
|  |  | Reverse： | CACAGAGAGCACAAGCAACTA |  |
| 8 | r-CD68 | Forward： | ACAAACAGTCCAGGCTTCTC | 126 |
|  |  | Reverse： | ACATGGCTGGGAACCATTAG |  |
| 9 | r-CD3 | Forward： | GAGAGCAGTCTGACAGATAGGA | 109 |
|  |  | Reverse： | CACCAGGAGGCTGAAGAATAAG |  |
| 10 | r-CD86 | Forward： | CTCAGTGATCGCCAACTTCA | 107 |
|  |  | Reverse： | GTTTCGGGTATCCTTGCTTAGA |  |
| 11 | r-CD206 | Forward： | CTTCTGTGCCTATCTCTCCAAC | 96 |
|  |  | Reverse： | GCCATGGCGACTTCAATTTC |  |
| 12 | r-IL-4ra | Forward： | AGTGGATGTGGAGGGTACAA | 87 |
|  |  | Reverse： | GTCCAGTCCGAAGGTGAATAAG |  |
| 13 | r-IL-6 | Forward： | GAAGTTAGAGTCACAGAAGGAGTG | 105 |
|  |  | Reverse： | GTTTGCCGAGTAGACCTCATAG |  |
| 14 | r-IL-10 | Forward： | AGTGGAGCAGGTGAAGAATG | 109 |
|  |  | Reverse： | GAGTGTCACGTAGGCTTCTATG |  |
| 15 | r-TNF-a | Forward： | GCAGATGGGCTGTACCTTATC | 101 |
|  |  | Reverse： | GAAATGGCAAATCGGCTGAC |  |
| 16 | r-iNOS2 | Forward： | CTACCAAGGTGACCTGAAAGAG | 82 |
|  |  | Reverse： | TGTTGTTGGGCTGGGAATAG |  |
| 17 | r-IL-1β | Forward： | GCAGATGGGCTGTACCTTATC | 101 |
|  |  | Reverse： | GAAATGGCAAATCGGCTGAC |  |

**Table S1.** Rat primer sequences used in real-time PCR analysis.


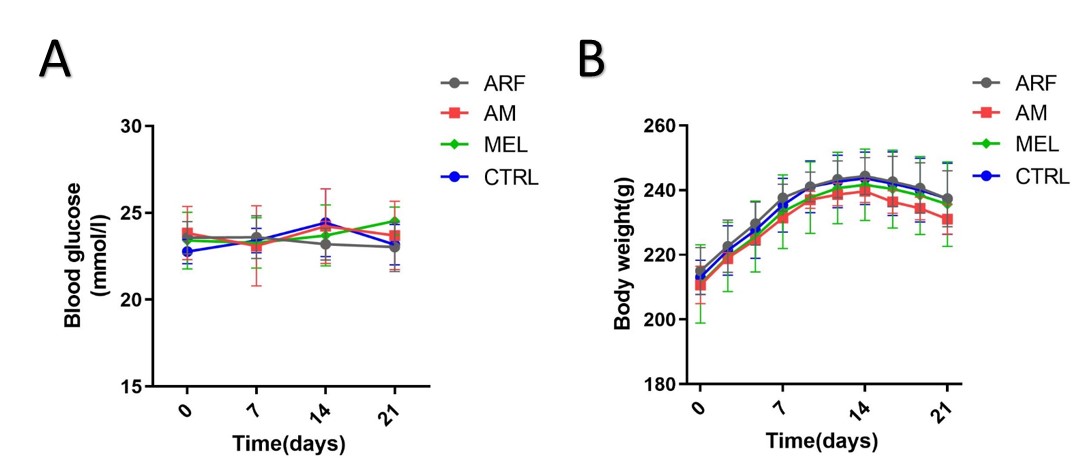


**Fig S1** (A) Quantification of the blood glucose over time after treatment. (B) Body weight changes of the mice over time after treatment in diabetic mice.


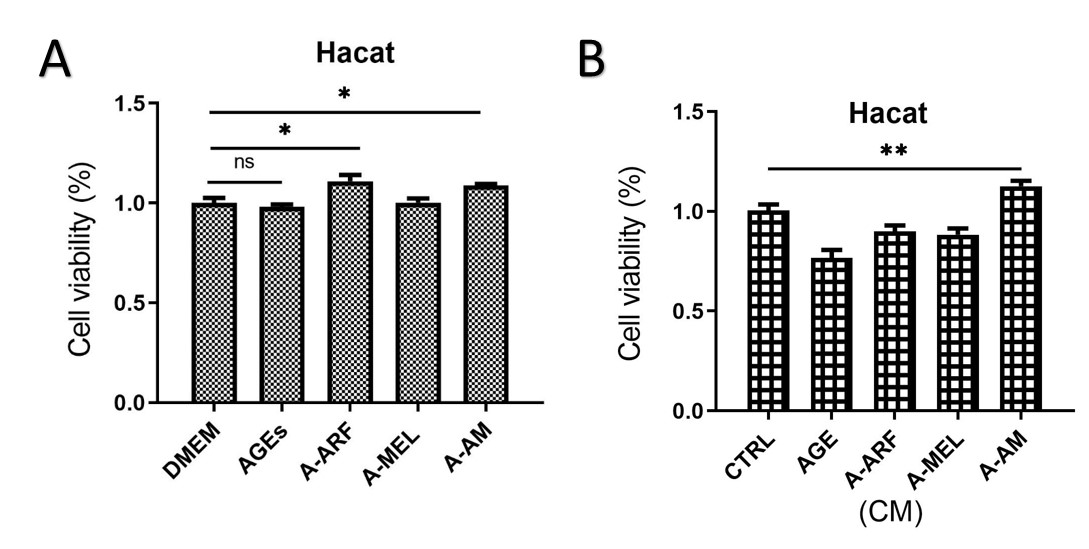


**Fig S2** (A) The proliferation rate of Hacat cultured in an AGE environment and treated with ARF and melatonin was analysed using the CCK-8 kit. (B) The proliferation rate of Hacat pretreated with different CMs was determined using the CCK-8 kit. Data were analysed using one-way ANOVA followed by Tukey’s multiple comparison test (A & B). *P < 0.05, **P < 0.01. n=5. Error bars represent SDs.


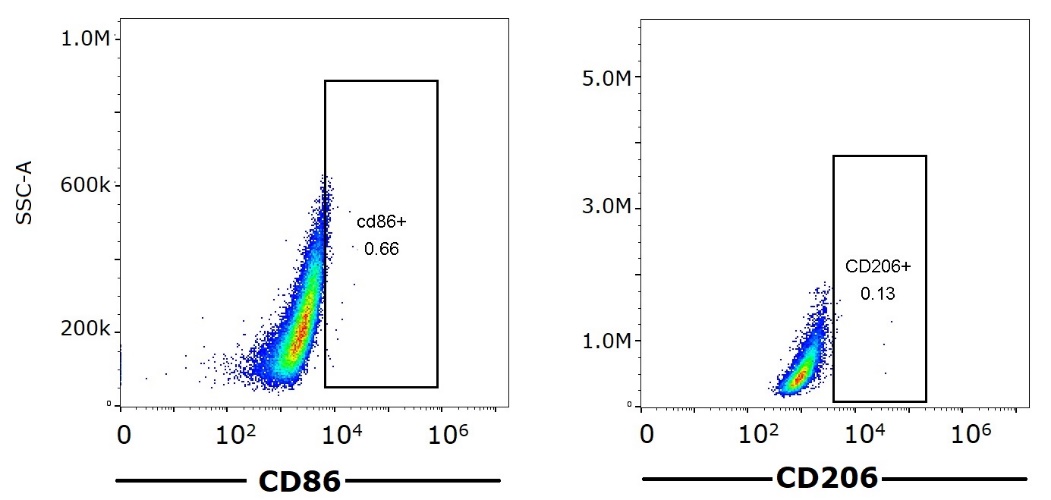


**Fig S3** Unstained cells (L929) were used for gating strategy of M1-like macrophage (CD86+ <1%) and M2-like macrophages (CD206+ <1%).
